# Yogurt Enriched with *Isochrysis galbana*: An Innovative Functional Food

**DOI:** 10.3390/foods10071458

**Published:** 2021-06-24

**Authors:** Joana Matos, Cláudia Afonso, Carlos Cardoso, Maria L. Serralheiro, Narcisa M. Bandarra

**Affiliations:** 1 Division of Aquaculture, Upgrading and Bioprospection (DivAV), Portuguese Institute for the Sea and Atmosphere (IPMA, I.P.), Avenida Alfredo Magalhães Ramalho, 6, 1495-165 Algés, Portugal; cafonso@ipma.pt (C.A.); carlos.cardoso@ipma.pt (C.C.); narcisa@ipma.pt (N.M.B.); 2 Faculty of Sciences, BioISI—Biosystems & Integrative Sciences Institute, University of Lisboa, Campo Grande 016, 1749-016 Lisboa, Portugal; mlserralheiro@fc.ul.pt; 3 CIIMAR, Interdisciplinary Centre of Marine and Environmental Research, University of Porto, Rua dos Bragas 289, 4050-123 Porto, Portugal

**Keywords:** *Isochrysis galbana*, ω3 long chain-polyunsaturated fatty acids, functional ingredient, yogurt, bioaccessibility

## Abstract

Microalgae are a valuable and innovative emerging source of natural nutrients and bioactive compounds that can be used as functional ingredients in order to increase the nutritional value of foods to improve human health and to prevent disease. The marine microalga *Isochrysis galbana* has great potential for the food industry as a functional ingredient, given its richness in ω3 long chain-polyunsaturated fatty acids (LC-PUFAs), with high contents of oleic, linoleic, alpha-linolenic acid (ALA), stearidonic, and docosahexaenoic (DHA) acids. This study focuses on the formulation of a functional food by the incorporation of 2% (*w*/*w*) of *I. galbana* freeze-dried biomass and 2% (*w*/*w*) of *I. galbana* ethyl acetate lipidic extract in solid natural yogurts preparation. In the functional yogurt enriched with microalgal biomass, the ω3 LC-PUFA’s content increased (to 60 mg/100 g *w*/*w*), specifically the DHA content (9.6 mg/100 g ww), and the ω3/ω6 ratio (augmented to 0.8). The in vitro digestion study showed a poor bioaccessibility of essential ω3 LC-PUFAs, wherein linoleic acid (18:2 ω6) presented a bioaccessibility inferior to 10% and no DHA or eicosapentaenoic acid (EPA) was detected in the bioaccessible fraction of the functional yogurts, thus indicating a low accessibility of lipids during digestion. Notwithstanding, when compared to the original yogurt, an added value novel functional yogurt with DHA and a higher ω3 LC-PUFAs content was obtained. The functional yogurt enriched with *I. galbana* can be considered important from a nutritional point of view and a suitable source of essential FAs in the human diet. However, this needs further confirmation, entailing additional investigation into bioavailability through in vivo assays.

## 1. Introduction

Nowadays, consumers have become more conscious about food ingredients, which has led to a growing demand for healthy natural products, and reinforced microalgae as an emerging and rich source of nutrients to be used in food supplementation [1]. There has been an increasing interest in ω3 long-chain (LC) polyunsaturated fatty acids (PUFAs) for nutritional and pharmaceutical applications. The nutritional importance of ω3 LC-PUFAs, mainly eicosapentaenoic acid (EPA, 20:5 ω3) and docosahexaenoic acid (DHA, 22:6 ω3), for human health is well established. Nevertheless, since humans cannot synthesize, in adequate levels, fatty acids with more than 18 carbons, they must be obtained from seafood, which is the major source of LC-PUFAs, particularly EPA and DHA [2]. Several studies have shown that EPA and DHA play an important role in the functional growth of brain cells, in preventing/reducing cardiovascular and inflammatory diseases, and also in preventing the progression of some types of cancer [1,2,3,4,5]. DHA is the predominant synaptosomal plasma membrane LC-PUFA in the brain, important for the normal neurological development. DHA has also been associated with positive effects on memory-related learning ability in Alzheimer’s disease [4,6].

LC-PUFAs constitute a large share of marine algal lipids, with planktonic algae being the source of most ω3FAs in fish [7]. There has been an increasing interest in microalgal lipids mainly because of their ability to synthesize high quantities of LC-PUFAs, as they are in fact the primary producers of ω3 LC-PUFAs, since they contain the necessary enzymes [8]. Microalgal lipids are divided into neutral lipids (triacylglycerols, diacylglycerols, and sterol esters), mainly located in lipid droplets in the cytoplasm or plastids, and polar lipids (phospho- and glycolipids), which build the fabric of cellular membranes [8]. The studied marine microalga *Isochrysis galbana* is a highly valuable source of natural bioactive compounds with important biological activities, such as hypocholesterolemic action [9]. The biomass of *I. galbana* is promising as a functional ingredient due to its considerably lipid content (20–30% dw) and richness of ω3 LC-PUFAs (mainly EPA and DHA) [2,10]. In addition, this marine microalga can provide highly valuable biological compounds, such as sterols, tocopherols, and fucoxanthin [2,9,11].

The change in dietary patterns in the human population, which has been particularly intense in the Western world, has led to an increase in ω6 FA consumption and a decrease in ω3 FA consumption, thus leading to an imbalance in the ω3/ω6 ratio level (desirably > 1). Very low ω3/ω6 ratios promote cardiovascular and inflammatory and autoimmune diseases, whereas increased levels of ω3 LC-PUFAs exert beneficial effects [12]. Given this background, efforts have been made to replace part of the vegetable or animal fat with marine lipids in foods such as mayonnaise, milk, bread, salad dressing, spreads, and yogurts [13]. Most food products have been prepared with fish oil, but, more recently, functional foods with a high content of algal ω3 LC-PUFAs have been tested, thus eliciting an industrial effort to produce such nutraceuticals. Moreover, the market for microalgae-containing foods has been expanding [14]. For instance, the microalgae *Arthrospira platensis, Chlorella vulgaris*, and *I. galbana* have been previously added as functional ingredients to biscuits [11,15], bread [16], and pasta [17].

Yogurt, one of the most consumed fermented dairy products in the world, is able to ensure the daily intake of nutrients and to bring positive impacts on consumers’ health due to its active cultures that promote healthy digestion and boost the immune system, providing health benefits [18]. Therefore, yogurt is an ideal vehicle to incorporate ω3 LC-PUFAs [19]. Dairy products such as yogurts have shown a high potential as carriers of microalgal biomass, ensuring a high share of microalgae ω3 LC-PUFAs and their bioaccessibility, given yogurt’s chemical and rheological properties, easiness to incorporate emulsions, and oxidative stability [3,20]. In fact, various yogurt products containing DHA have already been developed and marketed [19].

The aim of this work was to formulate a high-value functional food by the incorporation of freeze-dried biomass and ethyl acetate lipidic extract of *Isochrysis galbana* in commercial plain yogurt in order to increase ω3 LC-PUFAs content (mainly DHA) and to enhance ω3 LC-PUFAs bioaccessibility. Thus, based on the study’s results, the formulation of the functional yogurt could be optimized for maximal bioavailability of ω3 LC-PUFAs.

## 2. Material and Methods

### 2.1. Microalga Collection and Preparation

*Isochrysis galbana* biomass was supplied in freeze-dried form by Necton (Necton, Companhia Portuguesa de Culturas Marinhas, SA, Olhão, Portugal) and was analyzed without further processing.

### 2.2. Extract Preparation

A green solvent lipidic extraction from *I. galbana* freeze-dried biomass was performed. Briefly, 0.5 g of freeze-dried biomass was weighed, homogenized with 7.5 mL of ethyl acetate using a model Polytron PT 6100 homogenizer (Kinematica, Luzern, Switzerland) at a velocity of 20,000 rpm for 5 min, and agitated for 30 min on an orbital shaker at 300 rpm. After centrifugation (5000× *g* at 4 °C for 5 min), the organic phase was filtered through the anhydrous sodium sulfate column and evaporated in a vacuum rotary evaporator at 45 °C. Extractions were performed in quintuplicate. Samples were stored at −20 °C until further analysis.

#### Lipid Extraction Yield

For the lipid extraction from *I. galbana* freeze-dried biomass using the green solvent ethyl acetate, the FA extraction yield was calculated according the following equation:

(1) $$\mathrm{FA}\;\mathrm{extraction}\;\mathrm{yield}\;\left(\%\right)=\left(\frac{{\mathrm{FA}}_{f}}{{\mathrm{FA}}_{i}}\right)\times 100$$

where FA_f_ is the FA extracted from microalgal freeze-dried biomass (mg) using the solvent ethyl acetate and FA_i_ is the FA in the microalgal freeze-dried biomass (mg).

### 2.3. Functional Yogurt Preparation

The solid natural yogurts were purchased at a local store (Lisboa, Portugal). The yogurt labelled nutritional composition per 100 g was: 60 kcal, 3.5 g of lipids (among which 2.3 g of saturated fatty acids), 4 g of carbohydrates (4 g of which sugars), 3.2 g of proteins, and 0.13 g of salt. The yogurts were divided into three portions (125 g each), the first to prepare a control yogurt with no microalga addition, and the second and third portions to be used in the preparation of the functional yogurts. The incorporation level of *I. galbana* biomass in the yogurts was 2% (*w*/*w*) through the addition of freeze-dried microalga. This involved a gradual addition of the freeze-dried microalga with simultaneous mixture. The dried ethyl acetate lipidic extract was directly dissolved in the yogurt, at the same incorporation level of 2% (*w*/*w*), with simultaneous agitation to ensure a full homogenization of the extract. Therefore, 2.5 g of *I. galbana* freeze-dried biomass and dried ethyl acetate extract was added and dissolved in each yogurt (125 g), respectively. The yogurt samples were freeze-dried and stored at −80 °C until further analysis.

### 2.4. Nutritional Analyses

#### 2.4.1. Proximate Composition

The moisture and ash contents were determined according to the analytical protocol described in Association of Official Analytical Chemists [21]. For the moisture quantification, samples were dried overnight in an oven set to 105 °C, while ash was determined after combustion for 16 h at 550 °C. The protein level was quantified according to the Dumas method [22].

The total lipid content was determined following the Bligh and Dyer [23] extraction technique. Briefly, 5 mL methanol:chloroform (2:1), 1 mL of saturated NaCl solution and 2 mL of chloroform were added and homogenized with 100 mg of sample. After centrifugation (3000× *g* at 4 °C for 10 min), the organic phase was filtered through an anhydrous sodium sulfate column and evaporated in a vacuum rotary evaporator. Extractions were done in triplicate. Samples were stored at −20 °C until further analyses.

#### 2.4.2. Fatty Acid Profile

Fatty acid methyl esters (FAME) were prepared from the freeze-dried microalgal biomass, ethyl acetate extract, yogurt products and bioaccessible fractions by acid-catalyzed transesterification using the methodology described by Bandarra et al. [24]. FAME were applied to a DB-WAX (Agilent Technologies, Santa Clara, CA, USA) capillary column (film thickness, 0.25 μm), 30 m × 0.25 mm i.d., integrated in a Scion 456-GC gas chromatograph (West Lothian, UK), equipped with an autosampler with a split injector (100:1) and a flame ionization detector, both at 250 °C. FAME were identified by comparing their retention time with those of Sigma-Aldrich standard (PUFA-3, Menhaden oil). The limit of detection (LOD) is 1 mg/100 g. The results were expressed both in % of total fatty acids, as well as in mg/100 g of dry weight and wet weight basis using the lipid conversion factors set by a previous study. Analyses were performed in triplicate.

#### 2.4.3. Lipid Class

The amount of the different lipid classes was estimated by analytical thin-layer chromatography (TLC) following an optimized technique as described by Bandarra et al. [25]. The TLC was performed in plates coated with 0.25 mm silica gel G and developed with an eluent mixture of hexane:diethylether:acetic acid (65:35:1 by volume). The extracted lipids were dissolved in chloroform to a final concentration of 10 mg/mL. A mixture of standards (Sigma Chemical Co., St. Louis, MO, USA) was also prepared in chloroform with the same concentration, glyceryltrioleate (triacylglycerol, TAG), phosphatidylcholine (PC), oleic acid (free fatty acid, FFA), and cholesterol (CH) were used. The lipid class identification (polar and non-polar) was done by comparison with standards (TAG, PC, FFA and CH). The relative percentage of each lipid class was determined using a GS-800 densitometer and version 4.5.2 of Quantity One 1-D Analysis software from Bio-Rad (Hercules, CA, USA).

#### 2.4.4. Fatty Acid Composition of Polar and Non-Polar Lipids

The different lipid classes, phospholipids (polar lipids) and triacylglycerols (non-polar lipids), were isolated for fatty acid analysis in accordance with previously described procedures involving solid phase extraction and a preparative TLC [26]. This technique consisted of applying 25 μL of a microalga chloroform solution (in a concentration of 50 mg/mL) on several points of the TLC. The plate was placed in an elution vessel containing hexane:diethylether:acetic acid (65:35:1), and afterwards elution plates were sprayed with a 0.2% (*w*/*v*) solution of dichlorofluorescein (Sigma, St. Louis, MO, USA) in ethanol. Visualization was achieved under UV light at 254 nm in a cabinet II model UV chamber. The silica portions containing the lipid fractions were scratched from the plate and the FAME profile of the lipid classes was determined as in Section 2.4.2 Fatty acid profile.

### 2.5. In Vitro Digestion Model

An in vitro digestion model was chosen for the determination of FA bioaccessibility in *I. galbana* freeze-dried biomass and yogurt samples. The model simulates the human digestion in three different parts of the gastrointestinal (GI) tract: the mouth, stomach and small intestine. This is a static model very similar to the standardized model proposed by Minekus et al. [27]. This methodology involved preparing various digestive juices with appropriate mixtures of chemicals and enzymes. While chemicals were supplied by Merck (Darmstadt, Germany), enzymes were attained from Sigma (St. Louis, MO, USA). The solutions and enzymes used in this model were followed according Afonso et al. [28]. Approximately 0.5 g of *I. galbana* freeze-dried biomass or 1.5 g of yogurt (not freeze-dried) were weighed, taking into account the assumptions defined by Versantvoort et al. [29]. The mixture generated in the in vitro model was subjected to centrifugation at 2750× *g* for 5 min, thus yielding a non-digested portion and the bioaccessible fraction (supernatant attained in the centrifugation). The obtained fractions result from the effects of the digestive phenomena in each digestive compartment. Therefore, the small intestine bioaccessibility is the main result of the chosen model.

#### 2.5.1. Calculation of Bioaccessibility

The percentage (%) of total lipids (L) or each FA (FA) in the bioaccessible fraction was estimated as follows:

(2) $$\%\;L\;\mathrm{or}\;\mathrm{FA}\;\mathrm{bioaccessible}={\left[L\;\mathrm{or}\;\mathrm{FA}\right]}_{\mathrm{bioaccessible}}\times 100/\left[S\right]$$

where [L or FA] is the concentration of total lipids or FA; and [S] is the concentration of total lipids or fatty acids before digestion.

#### 2.5.2. Lipid Extraction in the Bioaccessible Fraction

For lipid extraction from the bioaccessible fraction, the Bligh and Dyer [23] method was slightly modified since the lipids were rendered available by the digestion procedure. First, it was added to the bioaccessible fraction 6 mL of chloroform followed by 1 min homogenization in a vortex and a centrifugation at 2000× *g* for 10 min at a temperature of 4 °C. Then, the organic phase was filtered through anhydrous sodium sulfate column. In the next step, 3 mL of chloroform was added followed by 1 min homogenization in a vortex and a centrifugation at 2000× *g* for 10 min at a temperature of 4 °C. The previous procedure was repeated. The organic phase was then evaporated. The lipid samples were weighed, solubilized in chloroform and stored at −80 °C until further analysis.

### 2.6. Statistical Analysis

To test the normality and the homogeneity of the variance of the data, Kolmogorov–Smirnov’s test and Levene’s F-test were used. Data which corroborated these assumptions were analyzed by one-way ANOVA distribution using the Tukey HSD post hoc test to determine the difference in the chemical composition between yogurt samples, in the lipid classes distribution between samples, in the fatty acid profiles between samples, and in the fatty acid composition between bioaccessible fractions. For all statistical tests, the significance level (α) was 0.05. All data analyses were performed using a statistical software, STATISTICA version 6.1 (Stat-sof, Inc., Tulsa, OK, USA, 2003).

## 3. Results and Discussion

**Figure 1:**
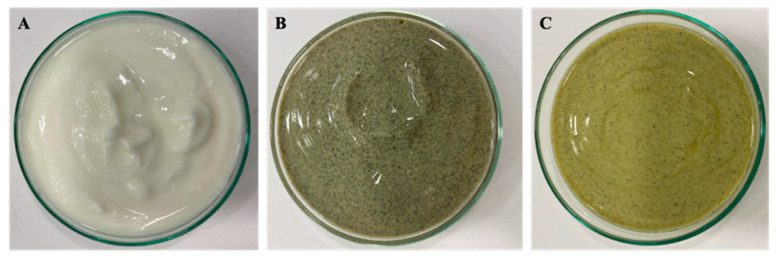
Yogurt products preparation: (**A**) Control Yogurt; (**B**) Yogurt with 2% (*w*/*w*) of *I. galbana* freeze-dried biomass; (**C**) Yogurt with 2% (*w*/*w*) of *I. galbana* ethyl acetate extract.

The microalga incorporation level of 2% (*w*/*w*) in the solid yogurts was chosen based on the literature available over microalgal biomass incorporation into food products [11,15,17], in order to not compromise the sensory acceptability of the final product in terms of color, fishy flavor and odor. The functional yogurts with 2% (*w*/*w*) of *I. galbana* of freeze-dried biomass and 2% (*w*/*w*) of *I. galbana* ethyl acetate extract incorporation presented an innovative green tonality (Figure 1).

The sensory attributes of the novel functional yogurt and the consumer’s acceptance need further evaluation.

### 3.1. Proximate Composition

The proximate composition of *I. galbana* freeze-dried biomass, *I. galbana* ethyl acetate lipidic extract, control yogurt and functional yogurts is presented in Table 1. The moisture content detected in *I. galbana* biomass was low (7.6 ± 0.1% dw), which was expected since the studied microalga biomass was freeze-dried. The dry matter of I. *galbana* was mainly composed of protein and lipids, 38.7 ± 0.0% dw and 24.5 ± 0.6% dw, respectively. The ash fraction was also a significant share of the biomass (14.6 ± 0.0% dw). The *I. galbana* ethyl acetate extract lipid content was 21.4 ± 0.9% dw. The observed proximate composition in the studied microalgal biomass is similar to that reported by other authors [10,17,30,31,32].

In the yogurt samples, the moisture content was approximately 88% ww and the ash fraction was 1% ww in all yogurts. The protein content in the yogurt enriched with *I. galbana* freeze-dried biomass was 4.0 ± 0.1% ww and about 3% ww in the control yogurt and yogurt with ethyl acetate extract. While the lipid content in control yogurt was 2.3 ± 0.3% ww, the lipid content was 2.7 ± 0.0% ww in yogurt with *I. galbana* freeze-dried biomass and 2.6 ± 0.1% ww in yogurt with ethyl acetate extract.

### 3.2. Lipid Classes

The lipid class distribution before and after digestion (bioaccessible fraction) of *I. galbana* freeze-dried biomass, *I. galbana* ethyl acetate lipidic extract, control yogurt and functional yogurts is presented in Table 2.

There were two main lipid classes in *I. galbana* biomass: triacylglycerols (TAG) with 36.8 ± 3.1% of total lipids and free fatty acids (FFA) with 32.6 ± 1.6% of total lipids. Polar lipids represented 14.8 ± 2.3% of total lipids. In *I. galbana* ethyl acetate extract, FFA were clearly the main lipid class, with 55.8 ± 0.1% of total lipids, followed by TAG with 18.1 ± 1.1% and polar lipids with 13.7 ± 0.0% of total lipids.

Previous studies on the microalga *I. galbana* reported high contents of FFA [10,32]. The authors Fidalgo et al. [30] concluded that the relative contents of each lipid class detected in this microalga may be explained by culture growth phase, being TAG content increased between exponential and stationary phases, and by variations in culture medium composition. Li et al. [33] reported that when microalgae are under nitrogen deprivation, a lipase is “activated” that acts specifically on glycolipids, hydrolyzing them, and the subsequently released FFA are incorporated into TAG. The same author suggested that TAG accumulation may be a way for the cell to prevent the reduction of molecular oxygen and consequent generation of ROS that are formed through photosynthesis, which are cytotoxic [33]. The small proportion of PL in *I. galbana* can be explained by the phase at which biomass was harvested, presenting marine microalgae species during the stationary phase an increase in TAG with a corresponding decrease in PL [30]. The limitation of nutrients for microalgae can be considered an important technique for FA and TAG production [34]. Microalgae growth can slow down under stress conditions, wherein the microalgal cells do not synthesize new membrane substances and FA are channeled to TAG synthesis as a mechanism of protection [34].

The main lipid class (as a percentage of the total lipid weight) present in the control yogurt was TAG with 47.1 ± 0.2% of total lipids. The same can be observed in the functional yogurts enriched with *I. galbana* freeze-dried biomass and yogurt enriched with ethyl acetate extract, with TAG being the main lipid class: 59.1 ± 0.3% and 52.8 ± 2.2% of total lipids, respectively. This result is in agreement with Paulo et al. [35], wherein TAG was also the main lipid class in yogurts enriched with the microalga *Aurantiochytrium* sp.

The level of lipid hydrolysis in the bioaccessible fraction can be evaluated by the distribution of lipid classes, as shown in Table 2. After in vitro digestion, FFA increased in the bioaccessible fraction when compared to the initial samples, by 21% in *I. galbana* freeze-dried biomass (reaching 53.0 ± 4.8% for FFA), by 21% in control yogurt (46.3 ± 1.8%), by 31% in yogurt with *I. galbana* freeze-dried biomass (44.7 ± 4.5%), and by 19% in the yogurt enriched with ethyl acetate extract (35.3 ± 2.0%). These results indicate a total TAG hydrolysis, with no TAG detected in the bioaccessible fraction, which agrees with previous studies with fish that claimed the same high level of TAG hydrolysis [26,28]. The increase in FFA in the *I. galbana* freeze-dried biomass bioaccessible fraction was also verified by Bonfanti et al. [10]. It should be noted that studies of microalgal lipid bioaccessibility using in vitro digestion model are still scarce [8,10,36].

### 3.3. Fatty Acid Profile

#### 3.3.1. Fatty Acid Profile of *I. galbana* Freeze-Dried Biomass and Ethyl Acetate Extract

The FA composition (in % of total FA and in mg/100 g dw) of the studied *I. galbana* freeze-dried biomass and ethyl acetate lipidic extract is shown in Table 3.

In *I. galbana* freeze-dried biomass, PUFAs were the main FA group with 47.9 ± 0.1% of the total FA, followed by the monounsaturated FA (MUFA) with 28.7 ± 0.0% and saturated FA (SFA) with 18.8 ± 0.7%. The profile was dominated by oleic acid (18:1 ω9), whose percentage exceeded 21% of total FA. Within the PUFAs group, ω3 LC-PUFAs were more abundant than ω6 LC-PUFAs, yielding an ω3/ω6 ratio of 2.2 ± 0.0. There were substantial levels of linoleic acid (18:2 ω6), α-linolenic acid (ALA, 18:3 ω3), stearidonic acid (18:4 ω3), and DHA, with proportions between 8 and 13%. EPA content was low, with approximately only 1% of total FA. In terms of FA concentrations in mg/100 g dw, oleic acid and linoleic acid were the most abundant, reaching 4015 ± 16 and 2260 ± 10 mg/100 g dw, respectively. The DHA content was 1637 ± 30 mg/100 g dw and EPA was 234 ± 1 mg/100 g dw.

**Figure 2:**
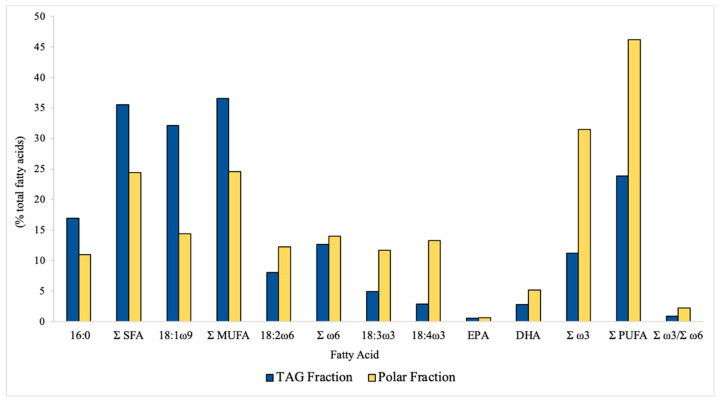
Fatty acid profile (in % of total fatty acids) of *I. galbana* TAG (triacylglycerol) and polar fractions.

For a more detailed analysis of *I. galbana* freeze-dried biomass FA profile, the composition of its main lipid classes was determined in the polar and TAG fractions. The FA composition (in % of total FA) of *I. galbana* polar and TAG fractions is presented in Figure 2. Whereas PUFAs were the main FA group (46.2% of total FAs) in the polar fraction, in the TAG fraction, MUFAs were the main FA group (36.5% of total FAs). In the polar fraction, ω3 LC-PUFAs were more abundant, yielding a ω3/ω6 ratio of 2.3. TAG fraction was poorer in ω3 LC-PUFAs, only yielding an ω3/ω6 ratio of 0.9. In both fractions, palmitic acid (16:0) was the most abundant SFA and 18:1 ω9 represented more than half of total MUFA. Significant contents of α-linolenic acid (18:3 ω3), stearidonic acid (18:4 ω3), and DHA were detected in the polar fraction within a range of 5–14%. Linoleic acid (18:2 ω6) represented almost all ω6 LC-PUFAs in the TAG fraction. EPA content was very low in both fractions (around 1%). DHA content was higher in the polar fraction (5.1%) than in the TAG fraction (2.8%), rendering the polar lipid fraction an interesting source of DHA.

It is important to note that the ω3 LC-PUFA composition, mainly EPA and DHA contents, present in the microalgae appear to be widely dependent on cultivation conditions, nutrient concentration, growth temperature, and growth phase at harvest time [10,30,37,38,39]. For example, various studies presented a higher FA accumulation in microalgae grown under nitrogen limitation [40,41,42,43]. The low EPA level detected in the present study was also observed by Bonfanti et al. [10] in the same microalga *I. galbana*. However, when comparing to other authors [11,17,30], detected EPA levels in the microalgae *I. galbana* are always higher than DHA levels, precisely the opposite of what was observed in the current study. The author Fradique et al. [17] detected an EPA level of 4875 mg/100 g dw in the same microalga *I. galbana*, a much higher level than in this study.

In the *I. galbana* ethyl acetate lipidic extract, PUFAs were the main FA group with 37.8 ± 0.5% of total FA, as in the microalgal biomass. Considerable levels of linoleic acid (18:2 ω6), ALA (18:3 ω3), stearidonic acid (18:4 ω3), and DHA were detected, with proportions within 6–10% of total FA. EPA content was also low, approximately 1% of total FA. Within the PUFA group, ω3 LC-PUFAs also exceeded ω6 LC-PUFAs, yielding an ω3/ω6 ratio of 2.1 ± 0.0. The ethyl acetate extract FA profile was dominated by the oleic acid (18:1 ω9) and myristic acid (14:0), reaching contents of 3501 ± 81 and 2446 ± 17 mg/100 g dw, respectively. As with the microalgal biomass, DHA content was higher (1021 ± 18 mg/100 g dw) when compared to EPA content (182 ± 28 mg/100 g dw).

The lipid extraction efficiency from the freeze-dried *I. galbana* with the green solvent ethyl acetate was approximately 19% (calculated on a dw basis). The FAs with the maximum extraction yield were myristic acid (14:0) and oleic acid (18:1 ω9) with 50% and 19%, respectively. LC-PUFAs presented the minimum extraction yield, wherein linoleic acid (18:2 ω6) and ALA (18:3 ω3) presented an extraction yield of 17% and stearidonic acid (18:4 ω3) of 15%. DHA and EPA presented an extraction yield of 13% and 17% dw, respectively. Therefore, the results show that the fatty acid extraction using the green solvent ethyl acetate could be more suitable for an extraction of SFAs.

The literature focused on *I. galbana* lipid extraction using the green solvent ethyl acetate is scarce and studies have been mainly focused on microalgal lipid extraction using traditional techniques (e.g., Bligh and Dyer, Soxhlet, Folch) and solvents (e.g., methanol/chloroform). Recently, the author Sánchez-Bayo et al. [44] also extracted lipids from the microalga *I. galbana* using the green solvent ethyl acetate with dried microalgal biomass, and the results show a similar lipid extraction yield of 18%. The same level of FAs group extraction yield was verified by Sánchez-Bayo et al. [44], wherein SFAs presented the maximum extraction yield and LC-PUFAs presented the minimum extraction yield. Ryckebosch et al. [45] used different solvent mixtures to extract lipids from *I. galbana*, with the lipid extraction being more efficient using the mixture chloroform/methanol (1:1, *v/v*) (28% yield) than hexane/isopropanol (3:2, *v/v*) (22%) and hexane (15%). Moreover, Wu et al. [46] used an eco-friendly solvent combination of methanol/ethyl acetate (2:1, *v/v*) for lipid extraction from the microalga *Chlorella* sp. and obtained a lipid extraction yield of 18%. Lipid extraction-assisted methods have been witnessing significant progress, such as microwave-assisted with different solvents, which achieved a maximum extraction yield from the microalga *Nannochloropsis* sp. of 52% with ethanol+acetic acid, 48% with methanol + hexane, and 37% with methanol [47].

Despite the lower lipid extraction efficiency achieved with ethyl acetate from *I. galbana* when compared to other solvent mixtures, this study was able to show the promising potential of this green solvent to extract microalgal lipids, as a way to replace the conventional toxic solvents with eco-friendly solvents for food industry application. Therefore, future research focused on extraction-assisted methods (such as ultrasound (US)-, microwave-, or enzyme-assisted extractions) with the green solvent ethyl acetate is required.

#### 3.3.2. Fatty Acid Profile of the Functional Yogurts

The fatty acid composition (in % of total fatty acids and in mg/100 g wet weight) of the control yogurt and functional yogurts is shown in Table 3.

SFA presented the highest share and displayed values between 60 and 64% of the total FA in control yogurt and functional yogurts. The same proportions in terms of abundances (SFA > MUFA > PUFA) in microalgae fortified yogurts were also verified by other authors [48,49]. PUFAs were the least abundant group of FAs, and ω6 PUFAs contents largely exceeded ω3 PUFAs, yielding an ω3/ω6 ratio lower than 1 in both functional yogurts. The incorporation of *I. galbana* freeze-dried biomass and ethyl acetate extract induced some differences when compared to the control, namely oleic acid, linoleic acid, and ALA contents were increased. Additionally, DHA and EPA contents were detected in both functional yogurts.

The ω3 LC-PUFAs content increased in yogurt with *I. galbana* freeze dried biomass, reaching 59 ± 4 mg/100 g ww. Oleic acid (18:1 ω9) was the most abundant FA in both functional yogurts (457 ± 16 mg/100 g ww and 418 ± 21 mg/100 g ww, respectively). Linoleic acid was the main ω6 LC-PUFA and stearidonic acid was the main ω3 LC-PUFA detected in both functional yogurts. DHA level detected in the yogurt with *I. galbana* freeze-dried biomass was 9.6 ± 1.0 mg/100 g ww, and in the yogurt with ethyl acetate extract was of 6.2 ± 0.6 mg/100 g ww. In comparison to the control, there was an increase in ω3/ω6 ratio (0.4 vs. 0.8) in the yogurt with *I. galbana* freeze-dried biomass, thus suggesting that functional yogurt enriched with *I. galbana* could be a relevant contributor to a more balanced diet.

The results agree with literature, wherein the microalgal supplementation increased ω3 LC-PUFAs levels (especially DHA and EPA) in feta cheese [50] and yogurts [35,51,52] and consequently decreased the ω6/ω3 ratio to a more beneficial level.

On the basis of the absolute FA composition, the yogurt with *I. galbana* freeze-dried biomass delivered 15.5 mg DHA + EPA/125 g serving, and the yogurt with *I. galbana* ethyl acetate extract delivered 10.9 mg DHA + EPA/125 g serving. The daily consumption of the functional yogurts enriched with the microalga *I. galbana* can only deliver a small portion of the recommended daily intake (RDI) of EPA + DHA suggested by the Food and Agriculture Organization (FAO) and the World Health Organization (WHO) of 250 mg/day EPA + DHA [53,54] and by the American Heart Association (AHA) of 500 mg/day for healthy adults [55,56]. A daily consumption of 125 g of this functional yogurt is not enough to fulfil dietary requirements, since a substantial number of functional yogurt servings (16 or 32 yogurt servings per day depending on FAO or WHO recommendations, respectively) would be needed every day to prevent cardiovascular disease. However, these functional yogurts enriched with *I. galbana* could still be a suitable daily source of ω3 LC-PUFAs in the human diet and can be considered nutritionally relevant. Of course, these results are conditional on the yogurt’s DHA+EPA being bioaccessible.

### 3.4. Fatty Acid Bioaccessibility

**Figure 3:**
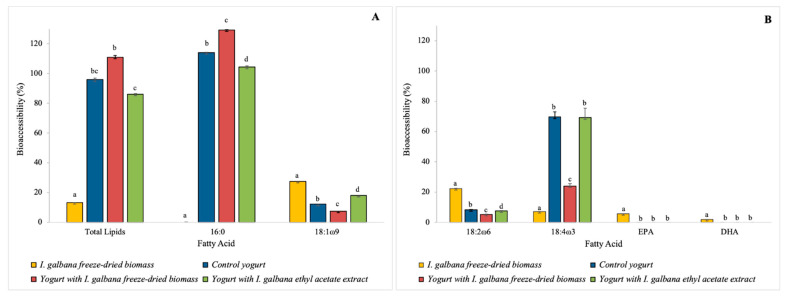
Bioaccessibility (%) of total lipids and specific fatty acids in the *I. galbana* freeze-dried biomass, control yogurt, yogurt with 2% (*w*/*w*) of *I. galbana* freeze-dried biomass and yogurt with 2% (*w*/*w*) of *I. galbana* ethyl acetate extract: (**A**) total lipids, palmitic and oleic FA; (**B**) polyunsaturated FA. Different lowercase letters correspond to statistical differences (*p* < 0.05) between the relative (%) FA of bioaccessible samples.

The specific bioaccessible FA (mg/100 g wet weight) in *I. galbana* freeze-dried biomass, control yogurt and functional yogurts are presented in Table 4. Additionally, the bioaccessibility percentages of total lipids and specific FA are shown in Figure 3.

The lipid bioaccessibility of *I. galbana* freeze-dried biomass was 13.2 ± 1.2%, indicating a low lipid availability for absorption at the intestine. This result is in agreement with Bonfanti et al. [10], who used the same digestion model for the same microalga. The highest bioaccessibility was detected for oleic acid (18:1 ω9), 27.5 ± 0.1%. DHA (22:6 ω3) was found to be barely bioaccessible (1.9 ± 0.0%) and EPA bioaccessibility was also low (5.7 ± 0.0%). The determined DHA and EPA bioaccessibility was lower than what Bonfanti et al. [10] reported.

The lipid bioaccessibility of the functional yogurts was high (exceeding 86%). In the control yogurt and both functional yogurts, the palmitic acid (16:0) was highly bioaccessible (>100%). Oleic acid (18:1 ω9) was more bioaccessible in the yogurt with ethyl acetate extract (18 ± 0.9%). Among the main FA, only palmitic acid (16:0) followed the pattern of bioaccessibility enhancement after *I. galbana* freeze-dried biomass and ethyl acetate extract incorporation in the yogurt matrix, which was also verified by Paulo et al.’s [35] study, which used the microalga *Aurantiochytrium* sp. in the development of a skimmed functional yogurt. Linoleic acid (18:2 ω6) showed a low bioaccessibility in all yogurts (<10%) and stearidonic acid (18:4 ω3) bioaccessibility was higher in the functional yogurt with ethyl acetate extract (69.2 ± 6.3%). No DHA or EPA content was found to be bioaccessible in the functional yogurts, despite their initial presence.

The observed bioaccessibility loss as a result of *I. galbana* incorporation is unexpected, since various studies considered yogurts an ideal food matrix for incorporating ω3 LC-PUFAs, making them more bioaccessible/bioavailable due to the preformed emulsions [3,57,58]. In view of the low bioaccessibility of ω3 LC-PUFAs, explanations must be found.

Though the direct use of microalgal biomass in nutrition has been advised owing to supposed high assimilation levels, there seems to be a higher difficulty in humans digesting microalgal biomass. The main critical difficulty in absorption is the microalgal cell wall, which is not degraded by the digestive enzymes present in the mouth, stomach, and small intestine. Therefore, since the absorption of nutritional compounds generally occurs in the small intestine, an intact microalgal cell wall may act as a natural physical barrier for (lipophilic) nutrients, thus limiting the digestibility of intracellular nutrients as well as the bioaccessibility of health-beneficial components [59].

The lower ω3 LC-PUFA bioaccessibility could result from a possible chemical interaction between lipids and other components from microalga. The microalgal FA released after hydrolysis may have a higher affinity for non-bioaccessible fractions components, such as polysaccharides present in the microalgae cell wall, or proteins and salts (such as calcium), establishing complexes and precipitating in the *pellet* (non-digested fraction) [8,10]. This was verified by Zhang et al. [60], who reported that some of the FA in the microalga *Chlorella* were attached to the cell wall and linked to carbohydrates by an ether bond. Therefore, since microalgal polar lipids are located in the cell membrane and in *I. galbana*, the main portion of DHA was found to be present in the polar fraction; this can explain why this ω3 LC-PUFA was not bioaccessible.

Since DHA and other ω3 LC-PUFAs are highly unsaturated, this may lead to a lower bioaccessibility percentage [10,26], which can also explain the low fatty acid bioaccessibility detected in this study.

A low lipid bioaccessibility means a lower nutritional value regarding ω3 LC-PUFAs content in the functional yogurts, but to achieve a higher level of bioaccessible LC-PUFAs, a much higher quantity of *I. galbana* freeze-dried biomass and ethyl acetate extract added to the yogurt than only 2% *w*/*w* would be needed. This would be unfeasible because of the impact on sensory properties. Therefore, it is important to find solutions to enhance the lipid bioaccessibility of microalgal ω3 LC-PUFAs for humans.

Some recent studies have used different techniques to increase ω3 LC-PUFAs contents and bioaccessibility. For instance, Señoráns et al. [61] used ultrasound-assisted extraction (UAE) with different temperatures and extraction times as an alternative method to the traditional lipid extraction techniques from the microalga *I. galbana*. In fact, UAE is considered a simple, economical and eco-friendly technique, thereby increasing the purity of the final product [47,62]. The study performed by Bernaerts et al. [59] proved the importance of cell disruption of the microalgae *Nannochloropsis* sp. for the in vitro lipid digestibility and bioaccessibility of ω3 LC-PUFAs, by using high pressure homogenization for cell disruption, which resulted in complete lipid digestibility and an increase in bioaccessibility. Prior to this, Cavonius et al. [36] subjected the microalga *Nannochloropsis oculata* to a pH-shift treatment process, which increased the accessibility of lipids after in vitro digestion. Enzyme-assisted lipid extraction techniques have also been reported [47,62]. Therefore, the formulation of functional food products enriched with microalgae biomass needs to consider a previous microalgal cell disruption.

## 4. Conclusions

The obtained results show the potential of incorporating the microalga *I. galbana* freeze-dried biomass as a functional ingredient into yogurts, wherein ω3 LC-PUFAs content was enhanced (60 mg/100 g ww), specifically concerning DHA (9.6 mg/100 g ww), and the ω3/ω6 ratio rose to 0.8. The incorporation of *I. galbana* freeze-dried biomass in yogurts was shown to be more effective in enhancing ω3 LC-PUFAs content (mainly DHA) than the ethyl acetate extract incorporation, which means that the green solvent lipid extraction from *I. galbana* was not as effective as expected. The in vitro digestion showed a poor bioaccessibility of ω3 LC-PUFAs, with no DHA or EPA present in the bioaccessible fractions, hence indicating a low lipid bioaccessibility. Nevertheless, when compared to the original yogurt, an added-value novel functional yogurt with DHA and a higher ω3 LC-PUFAs content was obtained, which can be considered important from a nutritional point of view and a suitable source of essential FAs in the human diet.

This study was able to prove the high potential of the microalga *I. galbana* as a functional ingredient, showing the importance of considering bioaccessibility in the evaluation of the nutritional value of microalgae-based functional foods, since microalgal bioactive compounds were poorly bioaccessible and only a small portion of the nutrients are ready for absorption. Therefore, future work and research are required to increase *I. galbana* lipid digestibility and enhance ω3 LC-PUFAs’ (mainly DHA and EPA) bioaccessibility/bioavailability to humans, with microalgae cell-disruption pretreatments (such as high-pressure homogenization or ultrasound-assisted extraction) being possible solutions. Moreover, for the viability of a future functional food, a sensory acceptability study will be fundamental.

## Figures and Tables

**Table 1:** Proximate composition (%) of *I. galbana* freeze-dried biomass, *I. galbana* ethyl acetate extract, control yogurt, yogurt with 2% (*w*/*w*) of *I. galbana* freeze-dried biomass, and yogurt with 2% (*w*/*w*) of *I. galbana* ethyl acetate extract.

Proximate Composition	*I. galbana* Freeze-Dried Biomass	*I. galbana* Ethyl Acetate Extract	Control Yogurt	Yogurt with *I. galbana* Freeze-Dried Biomass	Yogurt with *I. galbana* Ethyl Acetate Extract
	(% Dry Weight)	(% Dry Weight)	(% Wet Weight)	(% Wet Weight)	(% Wet Weight)
**Moisture**	7.6 ± 0.1	-	87.9 ± 0.1 ^a^	86.7 ± 0.0 ^b^	87.8 ± 0.1 ^a^
**Ash**	14.6 ± 0.0	-	0.7 ± 0.0 ^a^	1.0 ± 0.0 ^b^	0.7 ± 0.0 ^a^
**Protein**	38.7 ± 0.0	-	3.2 ± 0.1 ^a^	4.0 ± 0.1 ^b^	3.2 ± 0.1 ^a^
**Lipid**	24.5 ± 0.6	21.4 ± 0.9	2.3 ± 0.3 ^a^	2.7 ± 0.0 ^a^	2.6 ± 0.1 ^a^

Values are presented as average ± standard deviation. Different lowercase letters within a row correspond to significant differences (*p* < 0.05) between proximate composition of yogurt samples.

**Table 2:** Lipid class distribution (% of total lipid) before and after digestion (bioaccessible fraction) of *I. galbana* freeze-dried biomass, *I. galbana* ethyl acetate extract, control yogurt, yogurt with 2% (*w*/*w*) of *I. galbana* freeze-dried biomass and yogurt with 2% (*w*/*w*) of *I. galbana* ethyl acetate extract.

Sample		Lipid Classes			
		TAG ^1^	FFA ^2^	Polar Lipids	Sterol
***I. galbana* freeze-dried biomass**	Initial	36.8 ± 3.1 ^aA^	32.6 ± 1.6 ^aA^	14.8 ± 2.3 ^aA^	15.9 ± 0.9 ^aAB^
	Bioaccessible	nd ^b^^λ^	53.9 ± 4.8 ^b^^λ^	22.2 ± 2.6 ^b^^λ^	23.9 ± 5.1 ^a^^λ^
***I. galbana* ethyl acetate extract**	Initial	18.1 ± 1.1 ^B^	55.8 ± 0.1 ^B^	13.7 ± 0.0 ^A^	12.5 ± 1.2 ^A^
	Bioaccessible	-	-	-	-
**Control Yogurt**	Initial	47.1 ± 0.2 ^aC^	25.2 ± 0.3 ^aC^	12.7 ± 0.0 ^aAB^	15.0 ± 0.2 ^aA^
	Bioaccessible	nd ^b^^λ^	46.3 ± 1.8 ^b^^λ^	28.8 ± 1.6 ^b^^λ^	24.9 ± 0.2 ^b^^λ^
**Yogurt with *I. galbana* freeze-dried biomass**	Initial	59.1 ± 0.3 ^aC^	12.9 ± 1.1 ^aD^	20.2 ± 2.0 ^aAC^	7.8 ± 2.1 ^aAC^
	Bioaccessible	nd ^b^^λ^	44.7 ± 4.5 ^b^^λ^	32.1 ± 3.2 ^b^^φ^^λ^	23.2 ± 3.9 ^b^^λ^
**Yogurt with *I. galbana* ethyl acetate extract**	Initial	52.8 ± 2.2 ^aAC^	16.5 ± 2.3 ^aD^	18.0 ± 2.2 ^aAD^	12.8 ± 2.2 ^aA^
	Bioaccessible	nd ^b^^λ^	35.3 ± 2.0 ^b^^φ^	39.6 ± 5.8 ^b^^φ^	25.1 ± 4.9 ^b^^λ^

Values are presented as average ± standard deviation. nd—not detected. ^1^ TAG—triacylglycerol; ^2^ FFA—free fatty acid. For each sample, different lowercase letters within a column correspond to statistical differences between the initial and the bioaccessible samples (*p* < 0.05). For the initial samples, different uppercase letters within a column correspond to statistical differences between samples (*p* < 0.05). For the bioaccessible samples, different symbols within a column correspond to statistical differences between samples (*p* < 0.05).

**Table 3:** Fatty acid profile (in % of total fatty acids and in mg/100 g dry weight or wet weight) of *I. galbana* freeze-dried biomass and *I. galbana* ethyl acetate extract, of control yogurt, yogurt with 2% (*w*/*w*) of *I. galbana* freeze-dried biomass and yogurt with 2% (*w*/*w*) of *I. galbana* ethyl acetate extract.

Fatty Acid	*I. galbana* Freeze-Dried Biomass		*I. galbana* Ethyl Acetate Extract		Control Yogurt		Yogurt with *I.* galbana Freeze-Dried Biomass		Yogurt with *I. galbana* Ethyl Acetate Extract	
	% Total Fatty Acids	mg/100 g Dry Weight	% Total Fatty Acids	mg/100 g Dry Weight	% Total Fatty Acids	mg/100 g Wet Weight	% Total Fatty Acids	mg/100 g Wet Weight	% Total Fatty Acids	mg/100 g Wet Weight
14:0	5.5 ± 0.4 ^a^	1060 ± 66 ^A^	13.8 ± 0.1 ^b^	2446 ± 17 ^B^	12.6 ± 0.3 ^b^	229 ± 6 ^C^	12.3 ± 0.6 ^cb^	268 ± 13 ^C^	12.5 ± 0.4 ^b^	255 ± 9 ^C^
16:0	9.3 ± 0.5 ^a^	1777 ± 87 ^A^	11.3 ± 0.2 ^b^	1997 ± 37 ^B^	34.4 ± 0.3 ^c^	626 ± 5 ^C^	32.4 ± 0.3 ^d^	706 ± 7 ^C^	33.2 ± 0.1 ^e^	676 ± 3 ^C^
18:0	0.8 ± 0.0 ^a^	145 ± 0 ^A^	0.8 ± 0.0 ^a^	133 ± 3 ^A^	9.2 ± 0.3 ^b^	168 ± 5 ^B^	8.9 ± 0.3 ^b^	195 ± 7 ^C^	9.2 ± 0.2 ^b^	187± 4 ^C^
**Σ SFA ^1^**	18.8 ± 0.7 ^a^	3598 ± 137 ^A^	29.3 ± 0.5 ^b^	5183 ± 80 ^B^	63.8 ± 0.4 ^c^	1147 ± 3 ^C^	60.5 ± 0.8 ^d^	1319 ± 17 ^D^	62.1 ± 0.6 ^cd^	1264 ± 13 ^DC^
16:1 ω7	4.3 ± 0.0 ^a^	827 ± 7 ^A^	5.7 ± 0.0 ^b^	1004 ± 4 ^B^	1.8 ± 0.0 ^c^	33 ± 0 ^C^	2.2 ± 0.0 ^d^	48 ± 1 ^D^	2.0 ± 0.0 ^e^	41 ± 1 ^D^
18:1 ω9	21.0 ± 0.1 ^a^	4015 ± 16 ^A^	19.8 ± 0.5 ^a^	3501 ± 81 ^B^	19.9 ± 0.4 ^a^	362 ± 7 ^C^	21.0 ± 0.7 ^a^	457 ± 16 ^D^	20.5 ± 1.0 ^a^	418 ± 21 ^CD^
18:1 ω7	1.3 ± 0.0 ^a^	247 ± 3 ^A^	1.1 ± 0.0 ^ba^	189 ± 3 ^B^	2.0 ± 0.2 ^ca^	37 ± 4 ^C^	2.1 ± 0.1 ^ca^	46 ± 3 ^C^	1.8 ± 0.4 ^ca^	36 ± 9 ^C^
20:1 ω11	0.9 ± 0.1 ^a^	175 ± 9 ^A^	0.8 ± 0.0 ^a^	136 ± 1 ^B^	0.2 ± 0.1 ^b^	4.0 ± 1.7 ^C^	0.3 ± 0.0 ^b^	7.1 ± 0.5 ^C^	0.2 ± 0.1 ^b^	4.5 ± 1.9 ^C^
22:1 ω11	0.5 ± 0.0 ^a^	97 ± 3 ^A^	0.1 ± 0.0 ^b^	15 ± 0 ^B^	nd ^b^	nd ^B^	nd^b^	nd ^B^	nd ^b^	nd ^B^
**Σ MUFA ^2^**	28.7 ± 0.0 ^a^	5497± 8 ^A^	27.9 ± 0.4 ^a^	4932± 71 ^B^	24.0 ± 0.6 ^b^	401 ± 8 ^C^	25.7 ± 0.9 ^b^	560 ± 19 ^D^	24.7 ± 0.7 ^b^	502 ± 15 ^D^
16:2 ω4	0.3 ± 0.0 ^a^	57 ± 1 ^A^	0.4 ± 0.0 ^b^	65± 0 ^B^	0.2 ± 0.0 ^c^	2.9 ± 0.1 ^C^	0.2 ± 0.0 ^c^	3.4 ± 0.1 ^C^	0.2 ± 0.0 ^c^	3.4 ± 0.8 ^C^
18:2 ω6	12.1 ± 0.1 ^a^	2313 ± 14 ^A^	10.2 ± 0.2 ^b^	1806 ± 31 ^B^	2.2 ± 0.1 ^c^	40 ± 1 ^C^	3.0 ± 0.1 ^d^	65 ± 2 ^C^	2.6 ± 0.1 ^e^	54 ± 2 ^C^
18:3 ω3	11.8 ± 0.1 ^a^	2260 ± 10 ^A^	10.1 ± 0.1 ^b^	1779 ± 17 ^B^	0.6 ± 0.0 ^c^	11.5 ± 0.7 ^C^	1.4 ± 0.1 ^c^	30 ± 2 ^C^	0.8 ± 0.6 ^c^	16.9 ± 12.2 ^C^
20:4 ω3	0.2 ± 0.0 ^a^	35 ± 0 ^A^	0.2 ± 0.0 ^ab^	30 ± 0 ^AB^	nd ^b^	nd ^B^	nd ^b^	nd ^B^	nd ^b^	nd ^B^
20:4 ω6	0.3 ± 0.0 ^a^	66 ± 2 ^A^	0.2 ± 0.0 ^b^	39 ± 1 ^B^	0.1 ± 0.0 ^c^	2.0 ± 0.2 ^C^	0.1 ± 0.0 ^c^	2.8 ± 0.3 ^C^	0.1 ± 0.0 ^c^	2.7 ± 0.2 ^C^
18:4 ω3	10.2 ± 0.0 ^a^	1957 ± 3 ^A^	7.9 ± 0.2 ^b^	1387 ± 29 ^B^	0.6 ± 0.0 ^c^	10.1 ± 0.5 ^C^	0.8 ± 0.1 ^d^	16.7 ± 1.2^C^	0.5 ± 0.0 ^c^	9.4 ± 0.8 ^C^
20:5 ω3	1.2 ± 0.0 ^a^	234 ± 1 ^A^	1.0 ± 0.2 ^a^	182 ± 28 ^B^	nd ^b^	nd ^C^	0.1 ± 0.0 ^b^	2.8 ± 0.3 ^C^	0.1 ± 0.0 ^b^	2.5 ± 0.0 ^C^
22:5 ω3	0.2 ± 0.0 ^a^	35 ± 1 ^A^	0.1 ± 0.0 ^b^	22 ± 0 ^B^	nd ^b^	nd ^B^	nd ^b^	nd ^B^	nd ^b^	nd ^B^
22:5 ω6	1.9 ± 0.0 ^a^	358 ± 5 ^A^	1.2 ± 0.0 ^b^	220 ± 7 ^B^	nd ^c^	nd ^C^	nd ^c^	nd ^C^	nd ^c^	nd ^C^
22:6 ω3	8.6 ± 0.2 ^a^	1637 ± 30 ^A^	5.8 ± 0.1 ^b^	1021 ± 18 ^B^	nd ^c^	nd ^C^	0.4 ± 0.1 ^d^	9.6 ± 1.0 ^C^	0.3 ± 0.0 ^cd^	6.2 ± 0.6 ^C^
**Σ PUFA ^3^**	47.9 ± 0.1 ^a^	9154 ± 14 ^A^	37.8 ± 0.5 ^b^	6686 ± 90 ^B^	4.4 ± 0.1 ^c^	89 ± 2 ^C^	6.7 ± 0.2 ^d^	146 ± 7 ^C^	5.2 ± 0.4 ^c^	107 ± 8 ^C^
**Σ** **ω3**	32.5 ± 0.1 ^a^	6217 ± 16 ^A^	25.1 ± 0.1 ^b^	4426 ± 15 ^B^	1.2 ± 0.0 ^c^	22 ± 1 ^C^	2.7 ± 0.2 ^d^	60 ± 4 ^D^	1.6 ± 0.4 ^c^	32 ± 9 ^C^
**Σ** **ω6**	14.9 ± 0.0 ^a^	2847 ± 1 ^A^	12.2 ± 0.6 ^b^	2159 ± 104 ^B^	2.7 ± 0.1 ^c^	48 ± 2 ^C^	3.4 ± 0.1 ^d^	74 ± 1 ^C^	3.1 ± 0.1 ^c^	63 ± 2 ^C^
**Σ** **ω3/** **Σ** **ω6**	2.2 ± 0.0 ^a^	2.2 ± 0.0 ^A^	2.1 ± 0.1 ^a^	2.1 ± 0.1 ^A^	0.4 ± 0.0 ^b^	0.4 ± 0.0 ^B^	0.8 ± 0.1 ^c^	0.8 ± 0.1 ^C^	0.5 ± 0.1 ^b^	0.5 ± 0.1 ^B^

Values are presented as average ± standard deviation. nd—not detected. ^1^ SFA, saturated fatty acid; ^2^ MUFA, monounsaturated fatty acid; ^3^ PUFA, polyunsaturated fatty acid. Different lowercase letters within a row correspond to significant differences (*p* < 0.05) between the relative (%) FA profiles of samples. Different uppercase letters within a row correspond to significant differences (*p* < 0.05) between the absolute (mg/100 g dw or ww) FA profiles of samples.

**Table 4:** Specific bioaccessible fatty acids (mg/100 g wet weight) in *I. galbana* freeze-dried biomass, control yogurt, yogurt with 2% (*w*/*w*) of *I. galbana* freeze-dried biomass and yogurt with 2% (*w*/*w*) of *I. galbana* ethyl acetate extract.

Fatty Acid	*I. galbana* Freeze-Dried Biomass	Control Yogurt	Yogurt with *I. galbana* Freeze-Dried Biomass	Yogurt with *I. galbana* Ethyl Acetate Extract
	mg/100 g Wet Weight	mg/100 g Wet Weight	mg/100 g Wet Weight	mg/100 g Wet Weight
16:0	nd ^a^	714 ± 6 ^b^	912 ± 9 ^c^	706 ± 3 ^b^
16:1 ω7	256 ± 2 ^a^	15 ± 0 ^b^	2.3 ± 0.0 ^c^	nd ^d^
18:1 ω9	1105 ± 5 ^a^	44 ± 1 ^b^	34 ± 1 ^c^	75 ± 4 ^d^
18:1 ω7	54 ± 1 ^a^	8.2 ± 0.8 ^b^	4.8 ± 0.3 ^c^	7.8 ± 2.0 ^bc^
18:2 ω6	518 ± 3 ^a^	3.3 ± 0.1 ^b^	3.5 ± 0.1 ^b^	4.1 ± 0.1 ^b^
18:3 ω3	214 ± 1 ^a^	nd ^b^	nd ^b^	nd ^b^
18:4 ω3	139 ± 0 ^a^	7.0 ± 0.3 ^b^	4.0 ± 0.3 ^c^	6.5 ± 0.6 ^b^
20:4 ω6	44 ± 1 ^a^	nd ^b^	nd ^b^	nd ^b^
20:5 ω3	13± 0 ^a^	nd ^b^	nd ^b^	nd ^b^
22:6 ω3	32 ± 1 ^a^	nd ^b^	nd ^b^	nd ^b^

Values are presented as average ± standard deviation. nd—not detected. Different lowercase letters within a row correspond to statistical differences (*p* < 0.05) between the absolute (mg/100 g ww) FA of bioaccessible samples.

## Data Availability

The data presented in this study can be supplied on request.
